# Self-supervised domain adaptation of protein language model based solely on positive enzyme-reaction pairs

**DOI:** 10.1016/j.csbj.2025.11.045

**Published:** 2025-11-21

**Authors:** Tomoya Okuno, Naoaki Ono, Md. Altaf-Ul-Amin, Shigehiko Kanaya

**Affiliations:** a Graduate School of Science and Technology, Nara Institute of Science and Technology, Ikoma, 630-0192, Nara, Japan; b Department of Engineering Informatics, Osaka Electro-Communication University, Neyagawa, 572-8530, Osaka, Japan

**Keywords:** Protein language model, Enzyme-substrate interaction, Domain adaptation, Masked language modeling

## Abstract

There is growing interest in developing predictive models of enzyme catalytic properties that leverage activity data spanning diverse enzyme families. A fundamental challenge lies in the inherent biases of public biochemical databases. These databases predominantly catalog valid enzyme activities, rarely include negative instances, and report quantitative catalytic parameters for only a relatively small subset of enzymes. Such limitations pose a major obstacle to supervised learning of enzyme catalytic properties. One existing approach for model training involves generating synthetic negative enzyme-activity pairs by recombining existing enzymes and their activity information, particularly substrates or chemical reactions, that were not originally associated within datasets. However, it remains unclear whether the generated negative examples are truly inactive or merely unobserved active instances. To build a model that captures functional properties across diverse enzyme families while avoiding reliance on negative examples, this paper introduces a self-supervised domain adaptation methodology for pre-trained protein language models, solely based on positive enzyme-reaction pairs. The enzyme representations obtained from the adapted protein language model achieved superior or at least competitive performance compared to those from an existing method that relies on synthetic negatives, in both the turnover number prediction task for natural reactions of wild-type enzymes and the activity prediction task for family-wide enzyme-substrate specificity screening datasets. Overall, our approach represents a methodological advancement that eliminates the need for synthetic negatives and provides a scalable framework for leveraging the growing enzyme activity data in biochemical databases.

## Graphical Abstract

**Figure:**
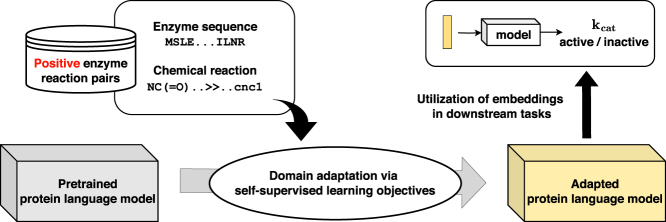

## 1 Introduction

Enzymes, which are proteins that function as biocatalysts to promote chemical reactions, have found widespread application due to their remarkable catalytic properties. Their advantages—namely mild reaction conditions, high product selectivity, and minimal environmental and physiological impact—have significantly contributed to the improvement of the efficiency and sustainability of commercial processes in a variety of fields, including food, biomedical, and environmental applications [1], [2], [3]. To elucidate enzyme function, considerable efforts have been devoted to using both experimental and computational approaches. As part of ongoing efforts, machine learning-based methods have recently emerged as a powerful complementary strategy to accelerate enzyme engineering, offering solutions for both the discovery and optimization stages [4].

Among the various applications of machine learning in enzyme engineering, the prediction of catalytic properties has garnered significant attention. Most existing models have been developed for specific enzyme families or a limited set of enzymes [5], [6], [7], [8], [9]. With the ongoing expansion of biochemical databases, there is growing interest in developing predictive models for enzyme catalytic properties that leverage enzyme activity data across a wide range of enzyme families [10], [11], [12], [13].

A fundamental challenge in harnessing such enzyme activity data for the development of models that capture enzyme functional properties across diverse enzyme families lies in the inherent bias of existing databases, which predominantly catalog only activities deemed valid, while those investigated but found invalid are often overlooked and remain significantly underrepresented. Additionally, quantitative catalytic parameters, such as turnover numbers, are reported for only a relatively small subset of enzymes. Such limitations hinder both classification due to the lack of negative examples and regression due to the scarcity of quantitative kinetic labels. An approach that has been reported to address these issues is the generation of synthetic negative enzyme-activity pairs by recombining existing enzymes and their activity information, particularly substrates or chemical reactions, that were not originally associated within datasets [10], [11]. This enables models to learn to distinguish between positive and negative examples.

Although the generation of negative examples provides a straightforward approach to addressing data imbalance, its validity remains a subject of ongoing debate. Specifically, there is uncertainty regarding whether the generated negative examples are genuinely inactive or simply unobserved active ones. Existing methodologies often operate under the assumption that all other combinations within datasets are inactive. While this assumption may hold approximately true for the relatively limited datasets used in previous studies, its applicability to larger and more complex datasets remains unclear. Moreover, the promiscuous activities exhibited by several enzymes raise additional concerns regarding their inclusion in such datasets [14].

To build a model that captures functional properties across diverse enzyme families while avoiding reliance on negative examples, this paper introduces a self-supervised domain adaptation methodology solely on positive enzyme-reaction pairs. While supervised training is often constrained by task-specific annotations, self-supervised learning fosters the acquisition of features that generalize across diverse applications. Specifically, our approach focuses on fine-tuning protein language models to capture sequence-function relationships of enzymes. A protein language model (PLM) is a language model pretrained on large and diverse protein sequence datasets, capable of producing embeddings for protein sequences. While the representations from protein language models capture general protein sequence patterns, further optimization is often required to adapt them to enzyme-specific tasks. For comparison with previous work, ESM-1b [15] was employed as a protein language model. Recent studies, such as RXNAAMapper [13], have applied self-supervised learning using a single unified model that processes amino-acid sequences and chemical reaction representations together, aligning sequence and reaction tokens to predict enzyme binding sites. In contrast, our approach employs a multi-encoder architecture, comprising distinct encoders for protein sequences and chemical reactions, where the pooled representation of the protein sequence is passed to the reaction encoder. This design encourages the PLM to concentrate information relevant to reaction masking into a single representational vector, which can be directly utilized as an encoder for amino-acid sequences in downstream enzyme-related tasks.

To evaluate the effectiveness of the output enzyme representations, both the turnover number prediction task for natural reactions of wild-type enzymes and the activity prediction task for family-wide enzyme-substrate specificity screening datasets are conducted. As a result, in both cases, the representations obtained from the adapted ESM-1b model achieved superior or at least competitive performance compared to those from the ESM-1b fine-tuned using the method of Kroll et al. [10], which relies on generated negative enzyme-substrate pairs.

## 2 Materials and methods

### 2.1 EnzSRP dataset

To fine-tune protein language models, the EnzSRP (enzyme amino acid sequence-reaction pair) dataset was constructed, comprising pairs of enzyme amino acid sequences and their associated enzymatic reactions, which were drawn from a diverse range of enzyme families. Following a similar approach to Hua et al. [11], the original data were collected from UniProtKB [16] and the Rhea database [17], which were linked through annotations [18]. Unlike previous methods that associate amino acid sequences and substrates through conventional classification frameworks, such as GO annotations and EC numbers [10], [13], this approach bypasses these intermediate steps, enabling a more direct mapping of sequences to reactions. The original data were downloaded via the UniProtKB web interface on 13 November 2024. Only entries from the Swiss-Prot section of UniProtKB with annotated catalytic activity were included. Swiss-Prot consists of manually curated and reviewed records, ensuring high-quality protein information. The evidence for catalytic activity annotations in Swiss-Prot includes both experimentally supported and non-experimental assertions. We did not restrict by evidence type and included all catalytic activity annotations. A total of 256,255 entries were retrieved, comprising 345,359 catalytic activity annotations.

These catalytic activity annotations were mapped onto the reaction SMILES format, which is an extension of the SMILES format for representing chemical reactions. This mapping process was performed using Rhea identifiers included in the catalytic activity annotations. Rhea identifiers listed under the “physiological reaction” field in catalytic activity annotations specify the direction in which the chemical reaction proceeds. In contrast, those listed under the “reaction” field represent primary identifiers without directional information. When directional information was available in the “physiological reaction” field, it was employed to define reactants and products. When only the “reaction” field was available, the reaction direction was derived from the MetaCyc database (version 27.0) [19]. As a result, 57,794 catalytic activity annotations without Rhea references and with undetermined reaction directions were excluded. Subsequently, the Rhea identifiers with directional information were mapped to MDL CT files (from the Rhea database; downloaded on 13 November 2024), and the mapped MDL CT files were converted to the reaction SMILES format using RDKit [20]. Bidirectional reactions, when identified, were treated as two independent reactions. Five Rhea identifiers that could not be mapped to any MDL CT files were excluded.

Amino acid sequences were preprocessed to handle cases involving isoforms and post-translationally processed chains, as described in the UniProtKB annotations. For the catalytic activity annotations associated with non-canonical sequences, specifically isoforms or processed chains, these non-canonical sequences were used, rather than the canonical sequences. Isoform sequences were obtained from UniParc (the UniProt archive; downloaded on 13 November 2024), while processed chains were derived from the canonical sequences by extracting the polypeptide chain ranges specified in the annotations.

Finally, a filtering process was applied based on both reaction types and tokenized sequence lengths. To comply with the pretrained model constraints, sequence-reaction pairs in which amino acid sequences exceeded 1022 tokens in length were removed. Additionally, pairs in which the chemical reaction sequences exceeded 446 tokens in length were excluded to reduce computational cost, because transformer-based models such as BERT employ attention mechanisms with $O(n^{2})$ complexity relative to sequence length. The tokenization methodology is described in Section 2.2. Reactions in which the reactants and products are identical, as determined from their reaction SMILES, were excluded. As stereochemical configuration is explicitly encoded in the SMILES representation, reactions involving inversion of chirality were retained in the dataset.

The resulting dataset, referred to as the EnzSRP dataset, comprises 310,391 enzyme-reaction pairs (Table 1). The combined proportion of EC 1 and EC 2 accounts for almost half of the total, and 10.8 % of the data has undefined EC numbers (Figure S1). The lengths of amino acid sequences in the dataset range from 11 to 1022 tokens, with a median length of 350 tokens, as shown in Figure S2. This uneven distribution suggests that certain enzyme families are substantially underrepresented, which could affect how domain adaptation captures functional diversity. We further discuss the potential implications of this dataset bias in Section 4.

**Table 1:** Summary of dataset sizes in previous datasets and EnzSRP dataset. A dagger (${\;}^{†}$) indicates the training split. ESP denotes final classification dataset, and ESP (pretrain)${\;}^{†}$ denotes the training set used for fine-tuning a protein language model [10]. EnzymeMap* denotes a derived version of EnzymeMap [22], first introduced by Heid et al. [21].

Dataset	N${\;}^{\circ }$ of pairs	N${\;}^{\circ }$ of enzymes	N${\;}^{\circ }$ of molecules
ESP [10]	69,337	11,940	1182
ESP (pretrain)${\;}^{†}$[10]	287,548	101,108	1274

			N${\;}^{\circ }$ of reactions

EnzymeMap* [21], [22]	46,356	12,749	16,776
ReactZyme [11]	178,463	178,327	7726
EnzSRP	310,391	169,112	10,256
EnzSRP${\;}^{†}$	278,248	151,613	9802

Finally, the EnzSRP dataset was clustered using CD-HIT [23] at an 80 % sequence identity threshold. To ensure that sequences from the same cluster were not split across subsets, the clusters were partitioned as whole units into training, validation, and test sets at proportions of 90 %, 5 %, and 5 %, respectively.

### 2.2 Chemical reaction tokenization

The following regular expression was utilized for chemical reaction tokenization.

(\%\([0-9]{3}\)|\[[^\]]+]|Br?|Cl?|N|O|S|P|F|I|b|c|n|o|s|p|\|| \(|\)|\ .|=|#|-|\+|\\|\/|:|~|@|\?|>>?|\*|\$|\%[0-9]{2}|[0-9])

This expression was originally designed to process both reaction SMILES and associated EC numbers in a single textual representation [24]. In this study, we use only reaction SMILES without EC numbers, and the expression remains fully applicable for handling reaction SMILES alone. A minimum frequency threshold of five was applied during vocabulary construction, with words occurring less frequently being excluded. As a result, the final vocabulary consisted of 124 items, including special tokens. The complete vocabulary used in the model is provided in the Supporting Information.

### 2.3 Self-supervised domain adaptation of protein language models

Domain adaptation refers to a type of transfer learning that addresses distribution shift by adapting models trained on a source domain to perform well on a different but related target domain [26], [27]. In this study, a pre-trained protein language model was fine-tuned on the EnzSRP dataset, which covers enzyme activities across a wide range of enzyme families. A protein language model is typically a self-supervised deep learning model that treats residues as tokens analogous to words in natural language. By pretraining on large and diverse protein sequence datasets, protein language models capture relationships among residues and produce residue-level contextual embeddings that capture general patterns in protein sequences. Here, ESM-1b was employed as the protein language model, a transformer-based architecture that outputs 1280-dimensional contextualized representations for each amino acid residue [15]. This choice of the model ensures consistency and enables direct comparison with the workflow presented in the study by Kroll et al. [10].

**Fig. 1:**
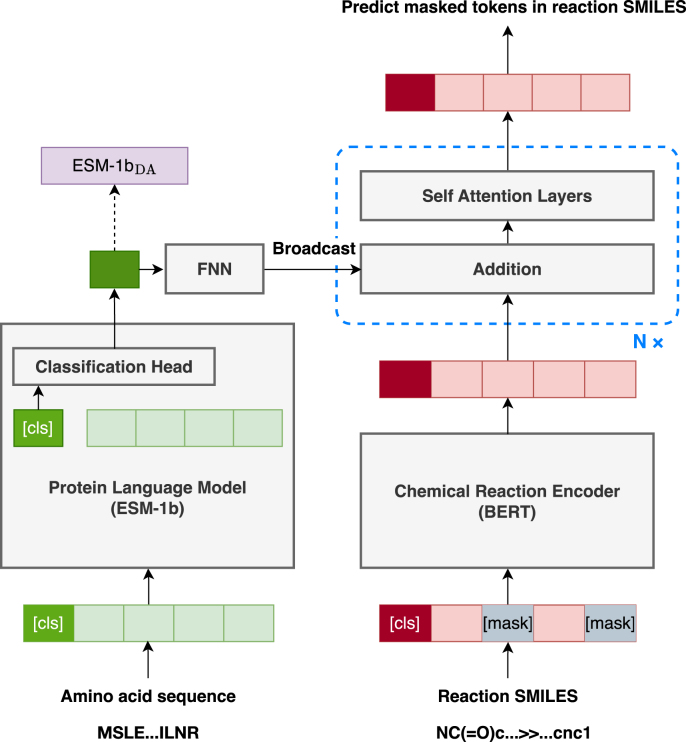
Model architecture for self-supervised domain adaptation of protein language model. This model primarily consists of two components: a protein language model, which serves as an encoder for amino acid sequences, and a chemical reaction encoder. The protein language model architecture follows the Hugging Face Transformers [25] implementation of ESM-1b without modification. The initial weights were loaded from the corresponding pretrained checkpoint. The final hidden state corresponding to the [CLS] token, which serves as a summary representation of the entire input sequence, is extracted, passed through both a pooling layer and a projection layer, and then uniformly added to the hidden states of all tokens in the chemical reaction sequence. The chemical reaction encoder is based on BERT and implemented using the Hugging Face Transformers library, with a modified configuration and custom parameters; it was randomly initialized and trained from scratch. After the addition of the amino acid sequence representation, the resulting hidden states are fed into subsequent self-attention layers, which are used to predict the masked tokens.

The protein language model was trained as part of a multi-encoder model that jointly processes amino acid sequences and chemical reactions (Fig. 1). The first encoder for amino acid sequence inputs employed the ESM-1b. To reduce computational overhead and mitigate overfitting, only the last four layers of the encoder were fine-tuned during training, while the remaining layers were kept frozen. The second encoder, used for chemical reaction inputs, employs the BERT architecture [28] due to its strong capacity for capturing contextual information and its proven performance across diverse natural language processing tasks. Accordingly, it serves as a robust baseline for evaluation. The encoder was implemented using the HuggingFace Transformers library [25] and was configured with custom model parameters in transformer depth, hidden size, and feed-forward dimensions. Specifically, it consists of two transformer layers, each with four attention heads, a hidden size of 512, and a feed-forward dimension of 2048. The protein representation produced by the protein language model is uniformly added to the hidden states of all tokens in the chemical reaction sequence. This simple additive fusion enables the model to incorporate protein information consistently across the entire reaction sequence, facilitating stable joint utilization of both modalities without introducing additional parameters. Finally, the resulting representations are passed through two BERT-style self-attention layers, which share the hidden size and feed-forward dimensions of the chemical reaction encoder.

To facilitate learning exclusively from positive examples, self-supervised training, particularly through masked language modeling applied to chemical reactions, was employed. Although a masking rate of 15 % is commonly employed in masked language modeling tasks, we adopt a higher rate of 30 % in our setting. This choice is motivated by the expectation that reactants and products exhibit a certain similarity, whereby a higher masking ratio is anticipated to increase training difficulty and thereby foster the acquisition of more robust representations. The model was optimized using the AdamW optimizer with $\beta_{1}=0.9$, $\beta_{2}=0.999$, and $\epsilon =1\times 10^{-8}$. Distinct learning rates were assigned: $5\times 10^{-5}$ for pretrained parameters and $2\times 10^{-4}$ for newly initialized parameters. To mitigate overfitting, a weight decay of 0.01 was applied. Gradient clipping with a maximum norm of 1.0 was employed to enhance training stability. No learning rate warm-up or scheduling strategies were incorporated. Furthermore, the input reaction SMILES were randomized in two ways: (i) alternative SMILES were generated for each molecule using RDKit, and (ii) the order of reactants and products was shuffled. These procedures were introduced to promote model generalization and to reduce overfitting to canonical representations.

Owing to GPU memory constraints, training was performed with gradient accumulation, whereby gradients are accumulated across multiple mini-batches before a single update, thereby simulating a larger batch size while reducing memory consumption. To further enhance computational efficiency, mixed-precision training with fp16 arithmetic (16-bit floating point) was employed, which accelerates training and improves memory efficiency. This approach appropriately balances fp16 and fp32 computations. Both gradient accumulation and mixed-precision training were implemented using the Accelerate library (version 0.33.0) [29].

Furthermore, owing to the high computational cost of training and evaluation, extensive hyperparameter optimization was not performed. All hyperparameters were initially fixed across experiments based on intuitive choices. Subsequently, to verify the robustness of these fixed settings rather than to tune them, a limited sensitivity analysis was conducted for key parameters (masking rate and learning rate), as described in Section 3.5.

Following the training phase, the ESM-1b component was extracted from the multi-encoder model and employed as the enzyme sequence encoder for subsequent downstream tasks. Hereafter, the enzyme representation adapted from ESM-1b outputs will be referred to as the ${\mathrm{ESM}\text{-}1b}_{\text{DA}}$ representation.

### 2.4 Turnover number prediction

To evaluate how well the ${\mathrm{ESM}\text{-}1b}_{\text{DA}}$ representation captures the semantic information of enzymatic activities, a turnover number prediction experiment was conducted, following the methodology proposed by Kroll et al. [12]. This approach employs a gradient boosting model to predict *in vitro* $k_{\text{cat}}$ values for the natural reactions of wild-type enzymes. The $k_{\text{cat}}$ measurement data used as the target values were collected from BRENDA [30], UniProtKB, and Sabio-RK [31].

Kroll et al. systematically evaluated multiple models for predicting enzyme turnover numbers, including models based exclusively on molecular features, models relying solely on enzyme sequence representations, and an ensemble model integrating both approaches. Among these, the ensemble model achieved the highest performance, as it combined predictions from a model utilizing protein sequence representations derived from a task-specific ESM-1b model [10] with those from a model based on differential reaction fingerprints (DRFPs). In this study, to evaluate both the intrinsic quality of the sequence representations and the upper bound of the achievable performance, we employed two categories of models: (1) models relying solely on protein sequence representations, and (2) ensemble models that integrate predictions from a model based on protein sequence representations with those from a model utilizing DRFPs.

### 2.5 Enzyme-substrate activity prediction

To assess the performance of the representations further, enzyme-substrate activity prediction experiments were conducted using five family-wide enzyme-substrate specificity screening datasets collected from the literature by Goldman et al. [9], covering halogenase [32], thiolase [6], β-keto acid cleavage enzymes (BKACE) [33], phosphatase [34], and esterase [35]. For comparison, we employed two types of representations: (1) the averaged token representations from the final layer of the ESM-1b model (hereafter ${\mathrm{ESM}\text{-}1b}_{\text{MEAN}}$ representations), and (2) the representations from the task-specific ESM-1b model fine-tuned by Kroll et al. [10] (hereafter ${\mathrm{ESM}\text{-}1b}_{\text{ESP}}$ representations). Since the training sets of the task-specific ESM-1b model [10] and our adapted ESM-1b model differ, we removed sequences that were highly similar to either of the training sets. This filtering procedure increases the difficulty of the prediction task but helps mitigate the potential confounding effect of training-test similarity on model evaluation. Sequence similarity for filtering was computed using the CD-HIT algorithm [23] with a similarity threshold of 60 %. The final filtered dataset is summarized in Table S1.

In the experiment, a neural network was employed as the predictive model (Figure S3). The input comprised two types of features: those derived from amino acid sequences and those associated with molecular structures. For the amino acid sequence features, the ${\mathrm{ESM}\text{-}1b}_{\text{MEAN}}$, ${\mathrm{ESM}\text{-}1b}_{\text{ESP}}$ and ${\mathrm{ESM}\text{-}1b}_{\text{DA}}$ representations were utilized. For the molecular features, the Morgan fingerprint (radius = 2, bit length = 1024) was adopted. Nested cross-validation was implemented for robust model evaluation, and the procedure was as follows. For each outer fold, hyperparameter tuning was performed using the corresponding inner training and validation sets. Based on the mean validation performance across the inner folds, the top-k hyperparameter configurations (with k=5 in our experiments) were selected. For each configuration, a final model was trained on the combined inner training and validation sets. These k final models were then aggregated into an ensemble to generate predictions on the outer test set. This process was repeated for all outer folds, and the resulting performance scores were averaged to obtain the final evaluation score. To ensure the reliability of the results, the entire nested cross-validation procedure was repeated ten times with different random seeds. The nested cross-validation employed four outer folds and three inner folds. For the outer data split, the enzyme discovery scenario, identifying new enzymes that can act on a known set of substrates [9], was used to evaluate extrapolation performance rather than interpolation capability. In contrast, the inner split was conducted using a simple random partitioning strategy, likely to enhance the stability of the hyperparameter tuning process. The area under the precision-recall curve (PR-AUC), calculated across all individual data points instead of on a per-sequence basis, served as the optimization and evaluation metric.

Hyperparameter optimization was performed using Optuna (version 3.4.0) [36]. A total of 50 optimization trials were conducted, comprising an initial random search phase of 10 trials. To account for differences in dataset size, the batch size was set to 32 for the thiolase dataset, 64 for the halogenase and β-keto acid cleavage enzymes (BKACE) datasets, and 512 for the esterase and phosphatase enzyme datasets. The maximum number of training steps was fixed at 2000 across all datasets, with validation performed every 50 steps during training. The search space for model hyperparameters was specified as follows. The number of hidden layers was fixed at either one or two. The dimensionality of each hidden layer was selected from integers ranging from 16 to 192, in increments of 16. The learning rate was sampled as a continuous value within the interval [0.0001, 0.02]. The dropout rate was similarly treated as a continuous variable ranging from 0.0 to 0.2. Weight decay, representing the strength of $\ell_{2}$ regularization, was sampled continuously within the interval [0.0001, 0.01].

### 2.6 Binding site prediction

To investigate the patterns captured by the pretrained model, we performed an attention analysis following the approach described in Teukam et al. [13]. In that study, a transformer-based masked language model, RXNAAMapper, was proposed to jointly encode enzymatic reaction SMILES strings and protein amino acid sequences as a concatenated input. Unlike structure-based approaches, RXNAAMapper identifies enzyme-substrate binding sites without relying on explicit three-dimensional structural information. The model was trained using masked language modeling (MLM) to capture contextual associations between reactant atoms and protein residues. Binding sites were predicted by computing atom-to-residue attention scores and selecting the top-k residues exhibiting the highest attention weights for each reactant atom. This procedure enabled the inference of putative catalytic or binding residues solely from sequence and reaction context.

In contrast to RXNAAMapper, our multi-encoder model processes amino acid sequences and chemical reactions in two separate encoders. Thus, there is no direct connectivity between protein sequence tokens and reaction tokens via the attention mechanism. Based on this distinction, we predicted binding sites solely based on the attention matrix of the adapted ESM-1b model, especially the attention matrix between [CLS] token and other amino acid sequence tokens in the last layer. This means that even different sequence-reaction pairs, if the sequence is identical, yield the same result. While this approach may present challenges when enzymes possess multiple catalytic functions, the limited dataset in the current investigation makes such issues unlikely to have a significant impact on the evaluation, as shown in Section 3.4. Note that the [CLS] token in the protein language model tends to aggregate information over the entire sequence that is informative for the reconstruction of the masked reaction; accordingly, attention from [CLS] to residue tokens is expected to focus on positions relevant for enzymatic function, making it a reasonable proxy for functional sites. These attention scores, however, should be interpreted with caution, as they likely reflect statistical associations rather than direct mechanistic relationships.

Additionally, to ensure a consistent comparison with RXNAAMapper, we constrain the number of predicted residues to match that used in RXNAAMapper. This constraint avoids confounding effects arising from differences in prediction coverage, which may otherwise artificially inflate the overlap score or diminish the apparent false positive rate. By fixing the prediction budget, we ensure that observed performance differences reflect model precision rather than variations in output quantity.

To quantitatively assess the accuracy of the predicted binding site residues, we employ two metrics: the overlap score (OS) and the false positive rate (FPR), as employed in Teukam et al. [13]. These metrics are defined as follows.

#### Overlap score (OS)

Let the predicted binding site region be denoted by $B={\left\{(a_{p_{i}},b_{p_{i}})\right\}}_{i=1}^{n}$, and the ground-truth region by $B_{s}={\left\{(a_{s_{j}},b_{s_{j}})\right\}}_{j=1}^{m}$, where $a_{i}$ and $b_{i}$ are the index boundaries of the segment i. The overlap score $\text{OS}(B,B_{s})$ is defined as:

(1) $$\text{OS}(B,B_{s})=\frac{\sum_{i=1}^{n}\sum_{j=1}^{m}\max\left(0,\min(b_{p_{i}},b_{s_{j}})-\max(a_{p_{i}},a_{s_{j}})\right)}{\sum_{i=1}^{m}(b_{s_{i}}-a_{s_{i}})}.$$

This metric quantifies the fraction of annotated binding site residues that are successfully recovered by the model’s predictions. A higher score indicates better coverage of the ground truth.

#### False positive rate (FPR)

The false positive rate (FPR) is defined as the proportion of predicted binding site residues that do not overlap with ground-truth binding site region. Formally, it is expressed as:

(2) $$\text{FPR}=\frac{\sum_{i}^{n}(b_{p_{i}}-a_{p_{i}})\cdot 1_{\overset{m}{\underset{j=1}{⋀}}\left([a_{p_{i}},b_{p_{i}}]\cap [a_{s_{j}},b_{s_{j}}]=\emptyset \right)}}{\sum_{i}^{n}(b_{p_{i}}-a_{p_{i}})}$$

Here, $1_{\overset{m}{\underset{j=1}{⋀}}(\cdot )}$ is an indicator function that evaluates to 1 if the predicted segment $\left[a_{p_{i}},b_{p_{i}}\right]$ has no overlap with any ground-truth segment $\left[a_{s_{j}},b_{s_{j}}\right]$ for all j∈{1,…,m}, and 0 otherwise. This metric quantifies the fraction of predicted residues that fall entirely outside ground-truth binding site region.

## 3 Results

### 3.1 Analysis of cluster structure in representation space based on EC numbers

To verify that the domain adaptation of ESM-1b improved the enzyme representations in a manner consistent with enzymatic functional organization, we conducted a preliminary evaluation of their cluster structure using EC number annotations and evaluated their agreement with the ground-truth enzymatic classification. Clustering metrics were computed to quantify the correspondence between the learned representations and the EC number-based labels. For this analysis, we used the EnzSRP test set, excluding entries without EC annotations. EC level 3 was employed for the label. EC level 3 classes represented by fewer than three sequences were omitted to ensure robust evaluation. The final dataset consisted of 9174 enzyme sequence-EC number pairs distributed across 141 distinct EC level 3 classes.

Table 2 summarizes the clustering performance using three standard evaluation metrics, including the Davies-Bouldin Index, Calinski-Harabasz Index, and the mean Silhouette score calculated as the average of per-cluster Silhouette scores, for both ${\mathrm{ESM}\text{-}1b}_{\text{MEAN}}$ and ${\mathrm{ESM}\text{-}1b}_{\text{DA}}$ representations. As a result, ${\mathrm{ESM}\text{-}1b}_{\text{DA}}$ representations show better scores in all the metrics than ${\mathrm{ESM}\text{-}1b}_{\text{MEAN}}$ representations, which do not consider any enzyme catalytic functions explicitly. Although the clustering scores are not sufficiently high for practical applications, our approach enables the learned representations to better reflect the EC number-based classification.

**Table 2:** Summary of clustering performance using three standard evaluation metrics: Davies-Bouldin index (DB Index), Calinski-Harabasz index (CH Index), and mean Silhouette Score computed per cluster. As a comparative baseline, ESM-1b${\;}_{\text{MEAN}}$ representation was used.

	DB index	CH index	Mean Silhouette (Cluster)
${\mathrm{ESM}\text{-}1b}_{\mathrm{MEAN}}$	3.23	18.5	0.0568
${\mathrm{ESM}\text{-}1b}_{\mathrm{DA}}$	2.09	40.3	0.263

### 3.2 Turnover number prediction

In the experiments conducted by Kroll et al. [12], the model with ${\mathrm{ESM}\text{-}1b}_{\text{ESP}}$ representations achieved slightly better performance than ${\mathrm{ESM}\text{-}1b}_{\text{MEAN}}$ representations, with ${\text{R}}^{2}=0.37$ and MSE=0.91 on the test set. The best-performing model, an ensemble of the ${\mathrm{ESM}\text{-}1b}_{\text{ESP}}$ model and the DRFP model, achieved a coefficient of determination of ${\text{R}}^{2}=0.44$ and MSE=0.81. In our study, we additionally evaluated a model with ${\mathrm{ESM}\text{-}1b}_{\text{DA}}$ representations, as well as an ensemble model that combines a model with ${\mathrm{ESM}\text{-}1b}_{\text{DA}}$ representations and one with DRFP.

**Fig. 2:**
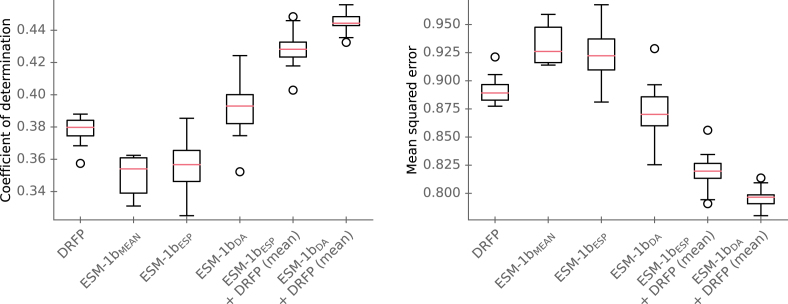
(left) Coefficients of determination ${\text{R}}^{2}$ for models with different inputs in the turnover number prediction experiment. (right) Mean squared errors (MSE) on $\log_{10}$-scale in the turnover number prediction experiment. Boxplots summarize the results on the test set using the optimized models with 10 different seeds. Model performances are plotted for the models using differential reaction fingerprints (DRFP), ${\mathrm{ESM}\text{-}1b}_{\text{MEAN}}$ representations, task-specific ESM-1b vectors (${\mathrm{ESM}\text{-}1b}_{\text{ESP}}$ representations) and ${\mathrm{ESM}\text{-}1b}_{\text{DA}}$ representations. Additionally, model performances were plotted for two ensemble models using ${\mathrm{ESM}\text{-}1b}_{\text{ESP}}$ representations and DRFP, as well as ${\mathrm{ESM}\text{-}1b}_{\text{DA}}$ representations and DRFP.

The predictive performances on the test set for ten models trained with different random seeds are presented in Fig. 2. ${\mathrm{ESM}\text{-}1b}_{\text{MEAN}}$ achieved a median $R^{2}=0.35$ and a median MSE=0.93, while ${\mathrm{ESM}\text{-}1b}_{\text{ESP}}$ showed a median $R^{2}=0.36$ and a median MSE=0.92, indicating similar performance according to the Wilcoxon signed-rank test (p>0.05). ${\mathrm{ESM}\text{-}1b}_{\text{DA}}$ obtained a median $R^{2}=0.39$ and a median MSE=0.87, outperforming both ${\mathrm{ESM}\text{-}1b}_{\text{MEAN}}$ and ${\mathrm{ESM}\text{-}1b}_{\text{ESP}}$ (p<0.05). For the ensemble models, ${\mathrm{ESM}\text{-}1b}_{\text{ESP}}$+DRFP model achieved a median $R^{2}=0.42$ and a median MSE=0.82, outperforming both DRFP and ${\mathrm{ESM}\text{-}1b}_{\text{ESP}}$ (p<0.05) as reported by Kroll et al. [12]. Our ${\mathrm{ESM}\text{-}1b}_{\text{DA}}$+DRFP model achieved a median $R^{2}=0.44$ and a median MSE=0.80, also outperforming ${\mathrm{ESM}\text{-}1b}_{\text{ESP}}$+DRFP model (p<0.05).

Kroll et al. reported that prediction quality tends to be higher for sequences that are more similar to those in the training set [12]. To further assess the representations, the test set was split according to the similarity score between the sequences in the test set and those in either the ESP training set used for fine-tuning or the EnzSRP training set. The similarity was computed using the CD-HIT algorithm, with a similarity threshold of 60 %. Even in low-similarity (60 %) subset, ${\mathrm{ESM}\text{-}1b}_{\text{DA}}$ representations showed higher performance than ${\mathrm{ESM}\text{-}1b}_{\text{ESP}}$ representations (Figures S4 and S5). Interestingly, the DRFP achieved higher absolute performance in the dissimilar subset, likely due to biases in dataset composition. The smaller performance gap between DRFP and the ensemble models in this subset likely reflects the reduced informativeness of enzyme-derived features, rather than a genuine improvement in the substrate representation.

To further mitigate potential bias arising from dataset composition, we performed an additional analysis to compare the molecular coverage between the two datasets (Figure S13). Specifically, for each molecule in the turnover-number prediction benchmark, we computed the maximum Tanimoto similarity of its ECFP4 fingerprint (Morgan radius = 2, 2048 bits) to molecules in the EnzSRP training set and in the ESP pretraining set. This analysis revealed that the ESP pretraining set more fully encompasses the chemical space of the turnover number prediction benchmark.

For completeness, we also conducted additional experiments using the ESM-2 model, the results of which are summarized in the Supporting Information (Figure S11).

### 3.3 Enzyme-substrate activity prediction

To examine the representational capacity of the ${\mathrm{ESM}\text{-}1b}_{\text{DA}}$ representations in the context of predicting catalytic activities for family-wide screens, we conducted activity prediction experiments on the β-keto acid cleavage enzymes (BKACE), esterase, halogenase, thiolase, and phosphatase datasets.

**Fig. 3:**
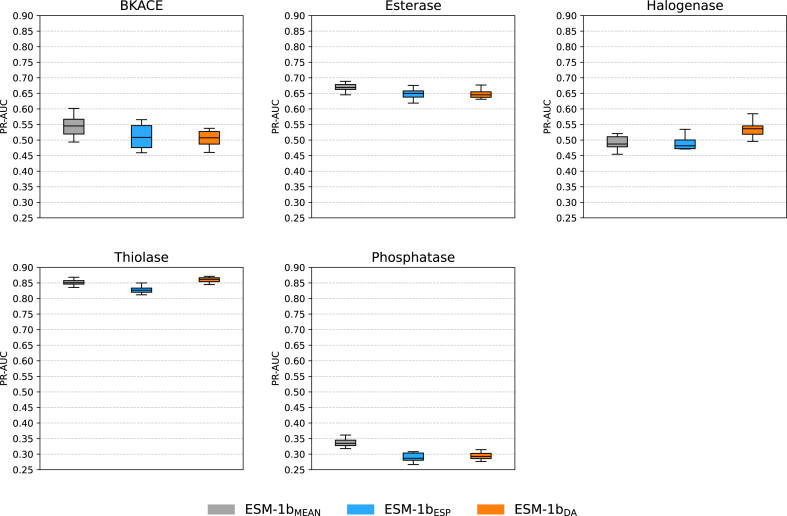
PR-AUC scores for three different sequence representations across five enzyme-substrate specificity screening datasets, obtained from 10 repetitions of nested cross-validation.

As illustrated in Fig. 3, ${\mathrm{ESM}\text{-}1b}_{\text{DA}}$ representations showed better performance than ${\mathrm{ESM}\text{-}1b}_{\text{ESP}}$ representations for the thiolase and halogenase datasets according to the Wilcoxon signed-rank test (p<0.05). For the BKACE, esterase and phosphatase datasets, ${\mathrm{ESM}\text{-}1b}_{\text{DA}}$ and ${\mathrm{ESM}\text{-}1b}_{\text{ESP}}$ representations showed comparable performance (p>0.05).

However, the performance of the ${\mathrm{ESM}\text{-}1b}_{\text{DA}}$ representations was lower than that of the ${\mathrm{ESM}\text{-}1b}_{\text{MEAN}}$ representations across the BKACE, esterase, and phosphatase datasets (p<0.05). To analyze this result further, we performed protein sequence functional annotation on both the EnzSRP training set and all enzyme activity screen datasets with InterProScan [37], [38]. InterPro entries found in the thiolase dataset are well-represented in the EnzSRP training set (Figure S6). For the halogenase dataset, although halogenases related to flavin-dependent halogenase are less represented in the EnzSRP training set, IPR036188 (FAD/NAD(P)-binding domain superfamily) is well represented (Figure S7). On the other hand, for the BKACE dataset, IPR008567, which represents the beta-keto acid cleavage enzyme, is found in only five sequences in the EnzSRP training set (Figure S8). Additionally, the esterase and phosphatase datasets encompass a broader set of InterPro entries compared to the other three datasets, and while several of these entries remain underrepresented in the EnzSRP training set (Figures S9 and S10). These underrepresented functional annotations in the training set may affect the results of catalytic activity classification. This outcome highlights an important challenge for models when making predictions on inputs outside the training distribution.

### 3.4 Binding site prediction

Teukam et al. [13] demonstrated that the best-performing layer in RXNAAMapper achieved an overlap score of 52.13 % and a false positive rate of 47.89 %. These results indicate that RXNAAMapper has a lower false positive rate than the Pfam-based method [39], as reported by Teukam et al. [13], which achieved an overlap score of 67.37 % and a false positive rate of 61.68 %.

In this experiment, we examined the attention weights in adapted ESM-1b and compared them with the outputs of RXNAAMapper. It is important to note that our model does not explicitly compute attention between the input sequence and the corresponding chemical reaction; therefore, attention scores between them cannot be directly analyzed. Instead, we analyzed the attention between the [CLS] token in the last layer of the adapted ESM-1b model, which summarizes the entire amino acid sequence, and the tokens corresponding to residues. Additionally, unlike RXNAAMapper, which selects the top-k tokens for each atom during score computation, we selected the same number of tokens as the outputs of RXNAAMapper for each enzyme-reaction pair to enable the performance comparison. While this approach does not allow absolute identification of binding sites solely based on our model, it is sufficient for evaluating relative performance.

For our adapted ESM-1b model, head 7 achieved the highest overlap score of 0.635 with a false positive rate of 0.473. This result demonstrates comparable performance to RXNAAMapper and suggests that our adapted ESM-1b model also has the potential to capture binding site patterns without three-dimensional structural information. Moreover, whereas the previous study employed Byte-Pair Encoding for tokenization, the present experiment demonstrated that comparable performance can be achieved without using it.

### 3.5 Sensitivity analysis of key hyperparameters

To assess the robustness of the model performance to key hyperparameters, we performed a sensitivity analysis on the masking rate and learning rate during domain adaptation, evaluating the resulting representations via the turnover number prediction task. As shown in Fig. S12, reducing the masking rate from 30 % to 15 % decreased the prediction performance to the point where it was no longer distinguishable from ${\mathrm{ESM}\text{-}1b}_{\text{ESP}}$ according to the Wilcoxon signed-rank test. In contrast, varying the learning rate resulted in performance that was comparable to or slightly higher than the original setting, and in both cases remained better than ${\mathrm{ESM}\text{-}1b}_{\text{ESP}}$. Detailed parameter settings are listed in Table S2.

## 4 Discussion

In this study, we introduced a self-supervised domain adaptation methodology for protein language models, which is based solely on positive enzyme-reaction pairs. The ESM-1b model was fine-tuned on the EnzSRP dataset, which comprises pairs of enzyme amino acid sequences and corresponding chemical reactions derived from a diverse range of enzyme families. In contrast to previous supervised methods and self-supervised frameworks employing unified sequence-reaction models, the ESM-1b model in our study was fine-tuned using a self-supervised multi-encoder architecture. This training scheme enables the model to derive a single representation vector that encapsulates enzyme-reaction-relevant information. We found that the ${\mathrm{ESM}\text{-}1b}_{\text{DA}}$ representations, which are domain-adapted outputs of the ESM-1b model, capture the nature of EC numbers on a subset of the EnzSRP test set. In the binding site prediction experiment, the observed overlap between attention hotspots and annotated binding sites highlights the adapted model’s potential to identify binding sites. It is important to note, though, that these attention scores should be regarded as indirect indicators rather than direct evidence of mechanistic relevance. In the turnover number prediction task for natural reactions of wild-type enzymes, these representations outperformed ${\mathrm{ESM}\text{-}1b}_{\text{ESP}}$ representations, which are the outputs of the task-specific ESM-1b model trained with synthetic negative pairs [10]. Additionally, the ${\mathrm{ESM}\text{-}1b}_{\text{DA}}$ representations demonstrate performance that is comparable to or exceeds that of ${\mathrm{ESM}\text{-}1b}_{\text{ESP}}$ representations in activity prediction for the family-wide enzyme-substrate specificity dataset. Although our evaluation scheme for predictive performance is limited to a comparison with a single task-specific supervised pretraining objective, we observe that the self-supervised representations retain more information relevant to enzyme function, with a clear effect in turnover number prediction. This pattern suggests that, within this experimental setting, self-supervised domain adaptation can produce representations that are less constrained by the pretraining objective. Overall, our methodology establishes a foundation for future studies, enabling them to leverage the growing volume of enzyme-related data in biochemical databases, which predominantly consist of positive enzyme activities.

A key limitation of our approach arises from the limited representational capacity of the learned embeddings. This limitation may be related to the compositional imbalance of the EnzSRP dataset, which could restrict the functional diversity encountered during pretraining. As indicated in Section 3.3, the resulting lower predictive performance on several screening datasets, compared to the mean-pooling representation, suggests that the model becomes biased toward dominant enzyme-reaction patterns. Addressing this issue will require adaptation procedures that explicitly counteract such imbalance, for example through regularization that limits representation drift or reweighting strategies that preserve diversity in catalytic functions.

Further limitations concern the restricted scope of hyperparameter tuning, which was constrained by the high computational cost of large-scale protein language models. In future work, employing more lightweight models could substantially reduce computational overhead while maintaining strong performance. Such an approach would make it feasible to conduct more comprehensive hyperparameter optimization, potentially leading to further improvements in model performance.

Finally, while the ESM-based representations in our model likely capture structural features learned from evolutionary patterns, our method does not explicitly incorporate structural information. Therefore, future integration of explicit structural data could further enhance the learned representations. Even in such cases, self-supervised training remains a promising strategy for mitigating the need to generate synthetic negative examples.

## Declaration of generative AI and AI-assisted technologies in the writing process

During the preparation of this work, the authors used ChatGPT (OpenAI) to improve English expressions, paraphrase sentences, and enhance the clarity of technical descriptions. After using this tool, the authors reviewed and edited the content as needed and take full responsibility for the content of the publication.

## Availability of data and materials

The code for EnzSRP dataset generation is available at the URL

https://github.com/motonuko/enzsrp. The code for turnover number prediction is available at the URL

https://github.com/motonuko/kcat_prediction_slim. The code for the other part of this work is available at the URL

https://github.com/motonuko/adaptplm. The EnzSRP dataset and results are available at the URL

https://doi.org/10.5281/zenodo.17015724.

## CRediT authorship contribution statement

**Tomoya Okuno:** Writing – review & editing, Writing – original draft, Visualization, Validation, Software, Project administration, Methodology, Investigation, Formal analysis, Data curation, Conceptualization. **Naoaki Ono:** Writing – review & editing, Supervision, Resources, Funding acquisition. **Md. Altaf-Ul-Amin:** Supervision. **Shigehiko Kanaya:** Supervision, Resources, Funding acquisition.

## Funding

This work was partially supported by 10.13039/501100001691JSPS KAKENHI Grant Numbers 23K05739 and 25K15934.

## Declaration of competing interest

The authors declare that they have no known competing financial interests or personal relationships that could have appeared to influence the work reported in this paper.
