# Supplementary material for: Self-supervised domain adaptation of protein language model based solely on positive enzyme-reaction pairs
Source: Comput Struct Biotechnol J. 2025 Nov 21;27:5441–9. doi: 10.1016/j.csbj.2025.11.045 (PMC12712682; doi:10.1016/j.csbj.2025.11.045)
Supplement: Multimedia Component 1 [file mmc1.pdf]

Supplementary Information
Self-supervised Domain Adaptation of Protein Language Model Based Solely on Positive Enzyme-reaction Pairs

Tomoya Okuno[a], Naoaki Ono[b], Md. Altaf-Ul-Amin[a], Shigehiko Kanaya[a]

[a] *Graduate School of Science and Technology, Nara Institute of Science and Technology, Ikoma, 630-0192, Nara, Japan*
[b] *Data Science Center, Nara Institute of Science and Technology, Ikoma, 630-0192, Nara, Japan*

Email address: okuno.tomoya.or9@naist.ac.jp (Tomoya Okuno)

Supplementary figures

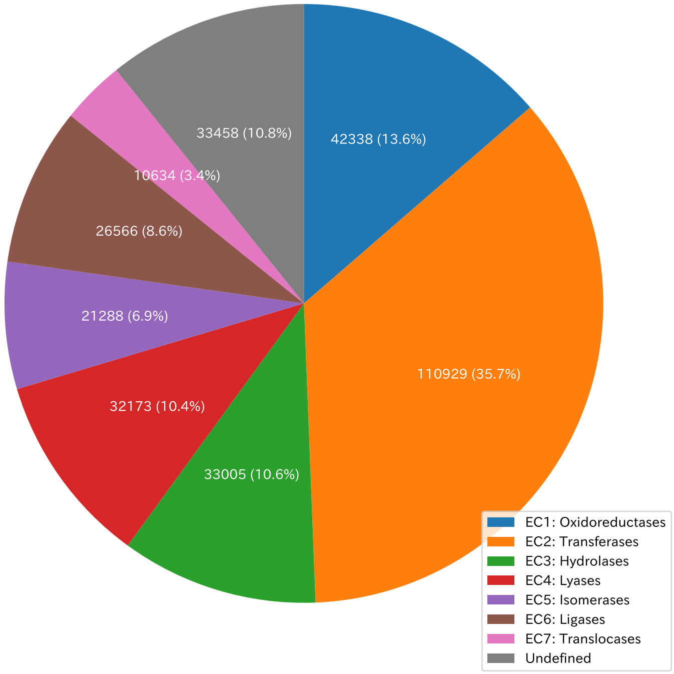

Figure S1: EC number distribution of EnzSRP dataset

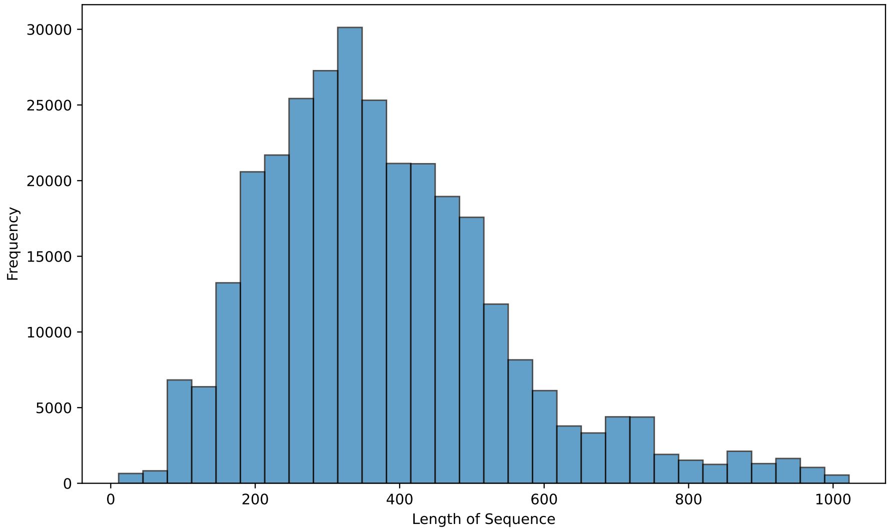

Figure S2: Sequence length distribution of EnzSRP dataset

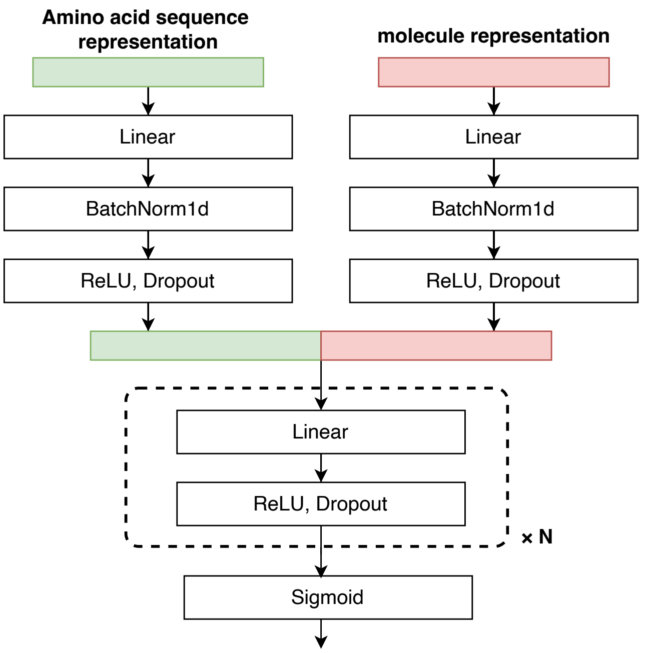

Figure S3: The model used in enzyme activity screening classification. N denotes the number of hidden layers following the concatenation of the amino acid sequence representation and molecular representation.

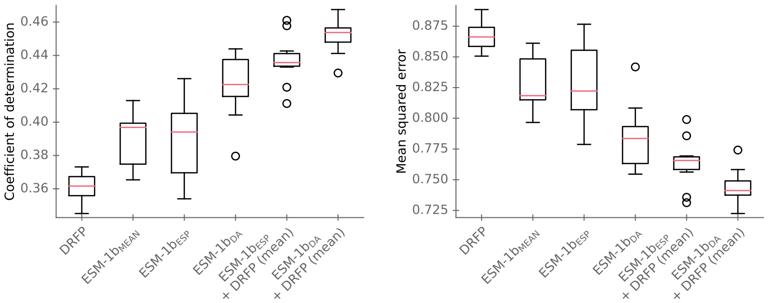

Figure S4: **(left)** Coefficients of determination R2 on similar sequences for models with different inputs in the turnover number prediction experiment (sequences similar to the EnzSRP training set or the ESP training set only). **(right)** Mean squared errors (MSE) on $\log_{10}$-scale in the turnover number prediction experiment (sequences similar to the EnzSRP training set or the ESP training set only). Boxplots summarize the results on the test set using the optimized models with 10 different seeds.

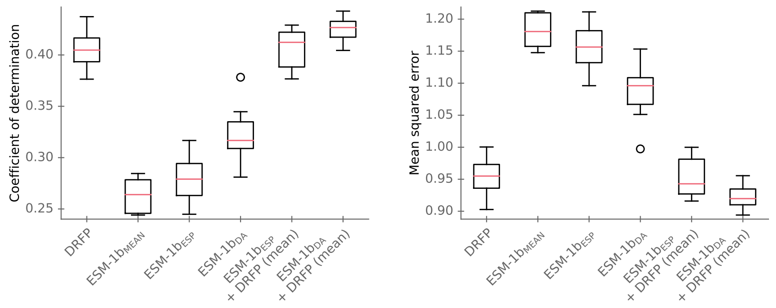

Figure S5: **(left)** Coefficients of determination R2 on unsimilar sequences for models with different inputs in the turnover number prediction experiment (sequences dissimilar to the EnzSRP training set or the ESP training set only). **(right)** Mean squared errors (MSE) on $\log_{10}$-scale in the turnover number prediction experiment (sequences dissimilar to the EnzSRP training set or the ESP training set only). Boxplots summarize the results on the test set using the optimized models with 10 different seeds.

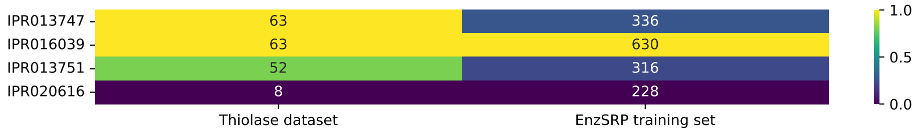

Figure S6: Number of sequences containing each InterPro entry in both the thiolase dataset and the EnzSRP training set. Each cell in the heatmap is colored based on its min-max normalized value (ranging from 0 to 1), while the annotated number indicates the corresponding raw value. Normalization is performed independently for each column.

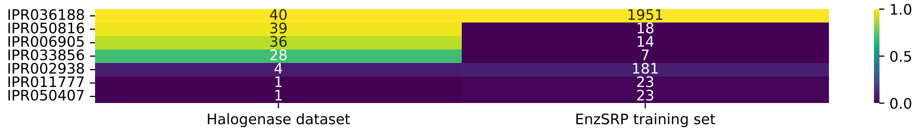

Figure S7: Number of sequences containing each InterPro entry in both the halogenase dataset and the EnzSRP training set. Each cell in the heatmap is colored based on its min-max normalized value (ranging from 0 to 1), while the annotated number indicates the corresponding raw value. Normalization is performed independently for each column.

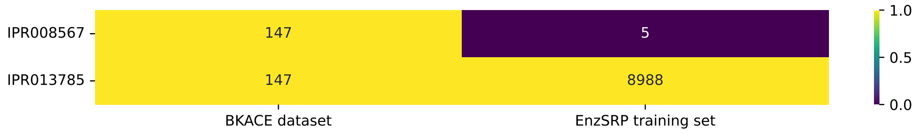

Figure S8: Number of sequences containing each InterPro entry in both the BKACE dataset and the EnzSRP training set. Each cell in the heatmap is colored based on its min-max normalized value (ranging from 0 to 1), while the annotated number indicates the corresponding raw value. Normalization is performed independently for each column.

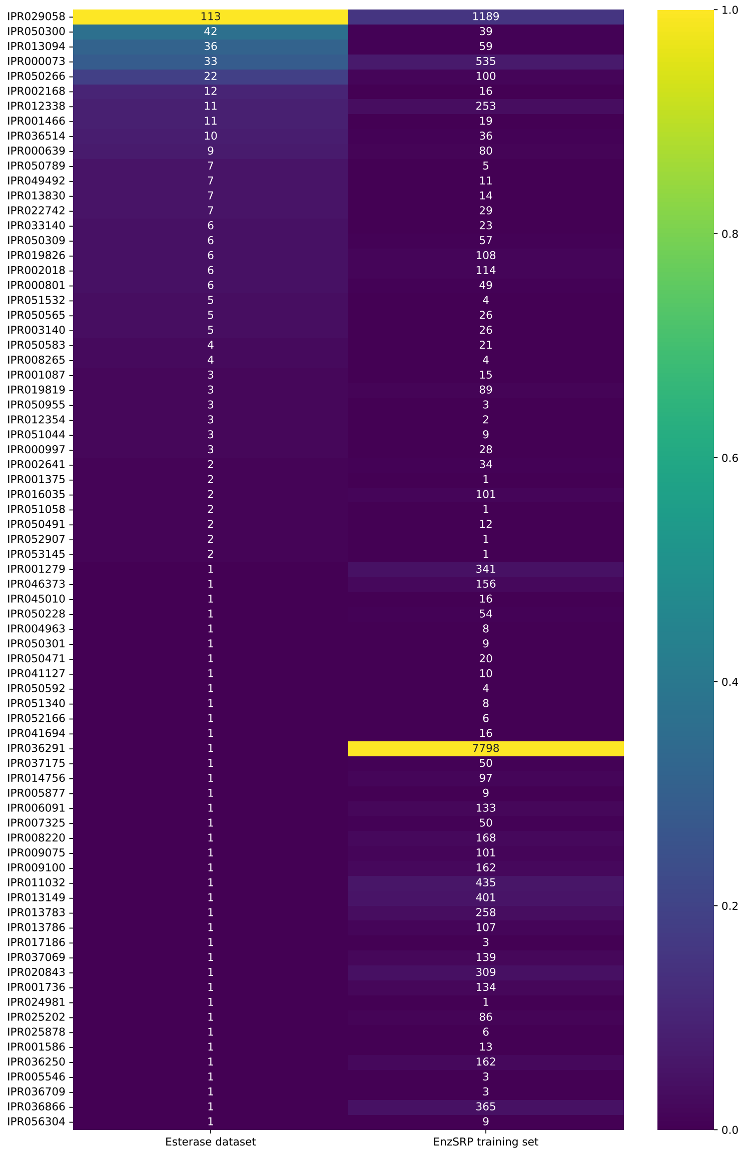

Figure S9: Number of sequences containing each InterPro entry in both the esterase dataset and the EnzSRP training set. Each cell in the heatmap is colored based on its min-max normalized value (ranging from 0 to 1), while the annotated number indicates the corresponding raw value. Normalization is performed independently for each column.

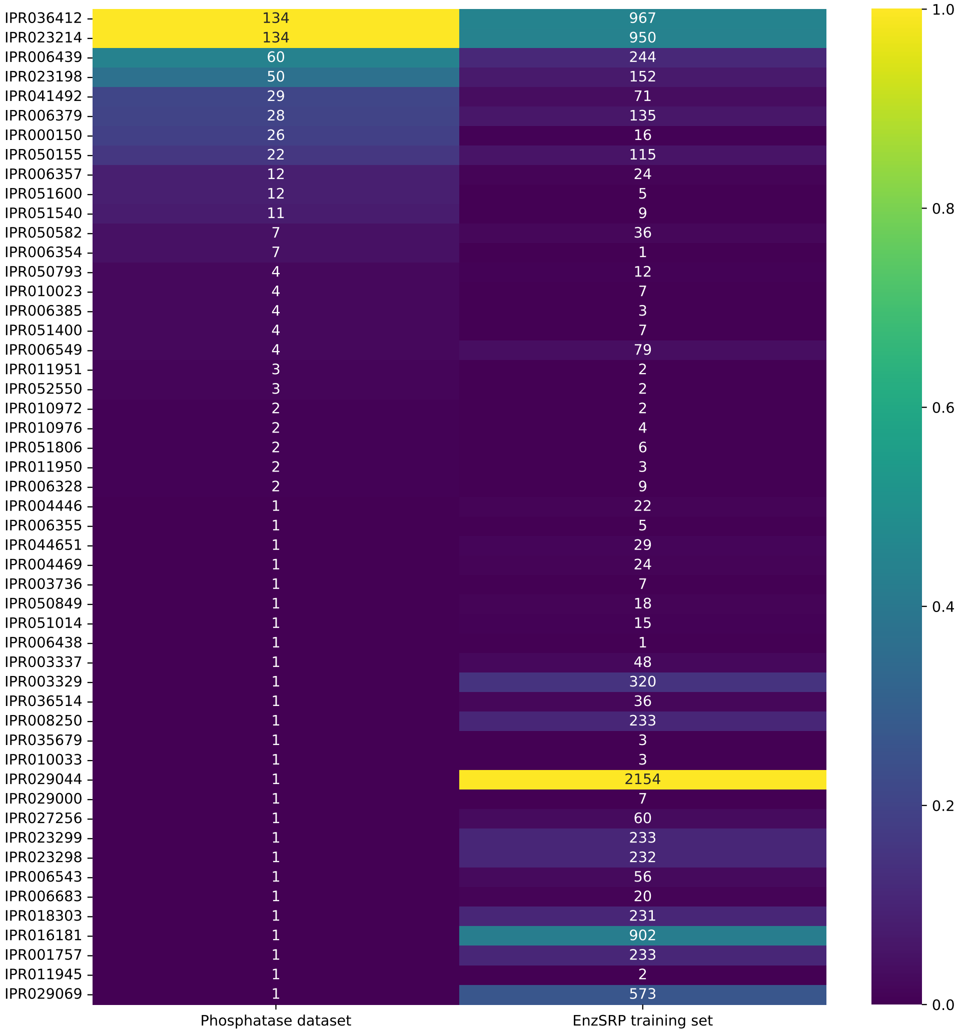

Figure S10: Number of sequences containing each InterPro entry in the phosphatase dataset and the EnzSRP training set. Each cell in the heatmap is colored based on its min-max normalized value (ranging from 0 to 1), while the annotated number indicates the corresponding raw value. Normalization is performed independently for each column.

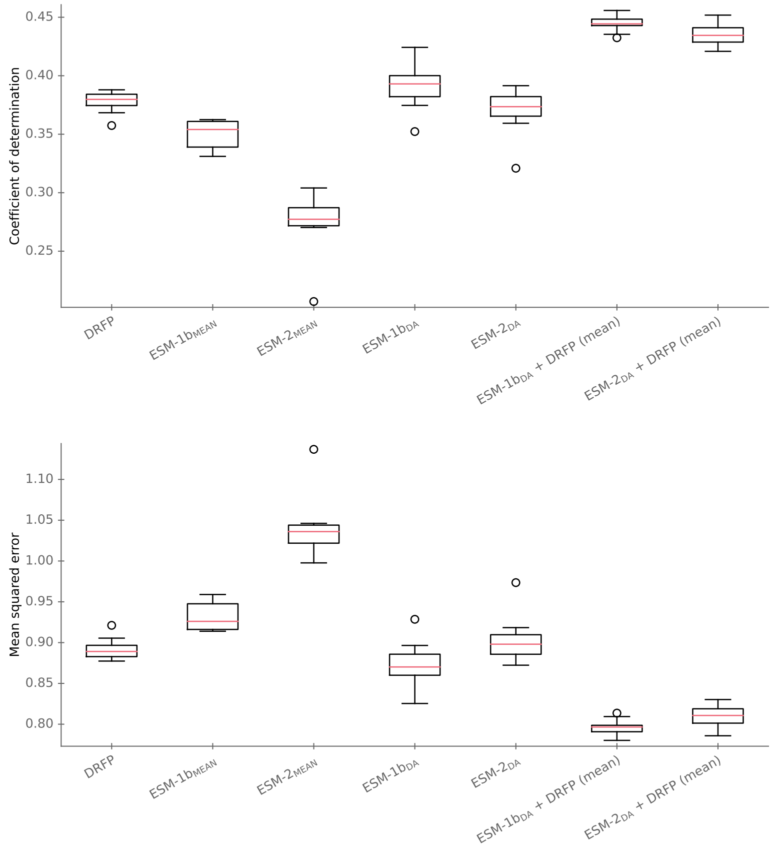

Figure S11: Results of the experiments using the ESM-2 (t33_650M_UR50D) model, which has approximately the same number of parameters as ESM-1b. **(Top)** Coefficients of determination (R^2). **(Bottom)** Mean squared errors (MSE) on $\log_{10}$-scale in the turnover number prediction experiment. Boxplots summarize the results on the test set using the optimized models with 10 different seeds. Interestingly, even without domain adaptation, ESM-2 showed slightly lower performance than ESM-1b on this task, no such difference was observed after domain adaptation. These results may stem from differences in how the two models represent enzyme-related sequences under the sequence-level embedding setup, where each sequence is represented by a single vector, rather than from their overall model capacity. Further investigation will be needed to clarify how specific architectural or pretraining differences influence sequence-level representations in enzyme-related prediction tasks.

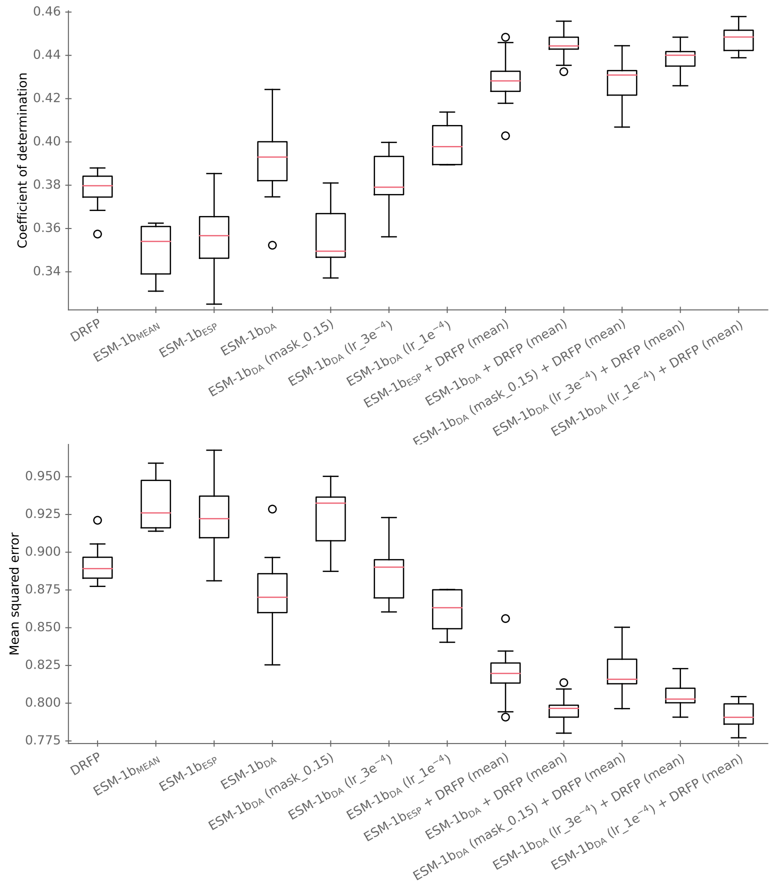

Figure S12: Results of the sensitivity analysis. Parameters are listed in Table S2. **(Top)** Coefficients of determination (R^2). **(Bottom)** Mean squared errors (MSE) on $\log_{10}$-scale in the turnover number prediction experiment. Boxplots summarize the results on the test set using the optimized models with 10 different seeds.

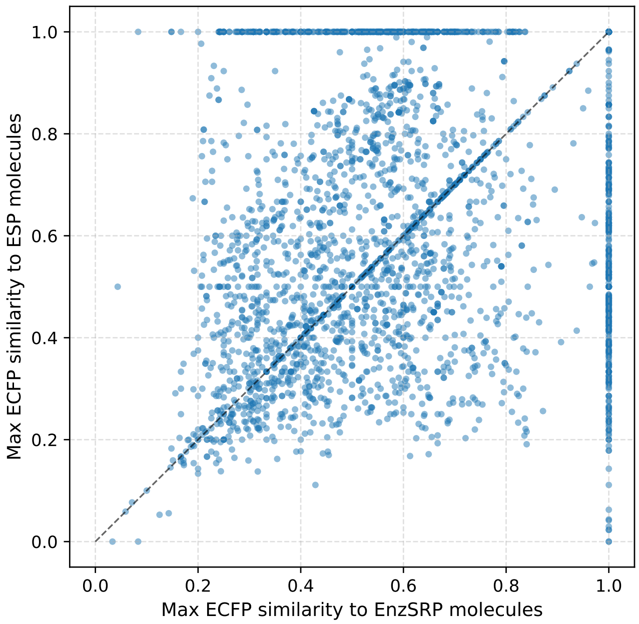

Figure S13: Comparison of molecular coverage between the EnzSRP training set and the ESP pretraining set. For each molecule in the turnover-number prediction benchmark, the maximum Tanimoto similarity based on its ECFP4 fingerprint (radius = 2, 2048 bits) was computed against molecules in the EnzSRP training set (x-axis) and the ESP pretraining set (y-axis). Each point represents a single molecule, with values bounded between 0 and 1. Molecules appearing below the diagonal line indicate higher maximum similarity to the EnzSRP training set, whereas those above the line indicate higher maximum similarity to the ESP pretraining set. Among 3,150 benchmark molecules, 1,160 (36.83%) had a higher maximum similarity to molecules in the EnzSRP training set, 1,378 (43.75%) had a higher maximum similarity to molecules in the ESP pretraining set, and 612 (19.43%) showed identical maximum similarity to both datasets. These results indicate that molecules in the ESP dataset more fully encompass the chemical space of the turnover-number prediction benchmark.

Supplementary tables

Table S1: Summary of activity screening datasets. Sequences that were close to either the ESP training set or the EnzSRP training set were filtered. The similarity boundary for this filtering was calculated using CD-HIT algorithm at a 60% threshold. The Glycosyltransferase dataset was not used in the experiments due to its small number of unique sequences.

dataset	**N° of pairs**	**N° of seq.**	**N° of mol.**
Halogenase [1]	2,480	40	62
Thiolase [2]	945	63	15
BKACE [3]	2,499	147	17
Phosphatase [4]	22,110	134	165
Esterase [5]	13,248	138	96
Glycosyltransferase [6]	905	11	90

Table S2: Parameters used in the sensitivity analysis. The learning rate values correspond to the *untrained* parts of the model, while the learning rate for the trained parts was kept fixed. Differences from the original setting are highlighted in bold.

	Mask rate	Learning rate	Training steps
original	0.30	2×10^{-4}	200,000
	**0.15**	2×10^{-4}	200,000
	0.30	$\mathbf{3 \times 10^{-4}}$	200,000
	0.30	$\mathbf{1 \times 10^{-4}}$	**300,000**

Vocabulary List

This section lists all tokens used in the model, excluding reserved tokens that are used for control or formatting purposes (e.g., sequence boundaries, padding, or unknown symbols).

```
 C I O P S ( ) F N 1 c 2 = O [O-] 3 n .  5 [C@H] [C@@H] 4 6 7 9
8 s | B [H] * :  ## >> [H+] - [NH3+] o [nH] / \ [C@] [C@@] [n+]
[Fe+] [N+] [S+] [1*] [NH2+] [Fe] [2*] [Fe+3] [SH] [Fe+2] [3*] [n-]
[4*] [Na+] [Fe-2] [nH+] [S-] [NH+] [C-] p [Se] Cl [Cu+] [Cu+2] [O+]
[Mo-] [Co-2] [c-] [*-] [K+] b [Fe-] [SeH] [Zn+2] [Mg-2] [Se-] [Mo]
[o+] [Ni+2] [Cl-] [Co+2] [As] [Ca+2] [I-] [Co+] Br [Mg+2] [Mn+2]
[Fe-3] [Cd+2] [Br-] [Ni-2] [N@+] [Mn+3] [Hg+2] [Ni-] [Hg] [W]
[Cr+6] [Cr+3] [F-] [cH-] [Hg+] [Co-4] [Co-3]
```

References

[1] B. F. Fisher, H. M. Snodgrass, K. A. Jones, M. C. Andorfer, J. C. Lewis, (2019), Site-selective c–h halogenation using flavin-dependent halogenases identified via family-wide activity profiling, ACS central science 5 1844–1856.

[2] S. L. Robinson, M. D. Smith, J. E. Richman, K. G. Aukema, L. P. Wackett, (2020), Machine learning-based prediction of activity and substrate specificity for olea enzymes in the thiolase superfamily, Synthetic Biology 5 ysaa004.

[3] K. Bastard, A. A. T. Smith, C. Vergne-Vaxelaire, A. Perret, A. Zaparucha, R. De Melo-Minardi, A. Mariage, M. Boutard, A. Debard, C. Lechaplais, et al., (2014), Revealing the hidden functional diversity of an enzyme family, Nature chemical biology 10 42–49.

[4] H. Huang, C. Pandya, C. Liu, N. F. Al-Obaidi, M. Wang, L. Zheng, S. Toews Keating, M. Aono, J. D. Love, B. Evans, et al., (2015), Panoramic view of a superfamily of phosphatases through substrate profiling, Proceedings of the National Academy of Sciences 112 E1974–E1983.

[5] M. Martínez-Martínez, C. Coscolín, G. Santiago, J. Chow, P. J. Stogios, R. Bargiela, C. Gertler, J. Navarro-Fernández, A. Bollinger, S. Thies, et al., (2017), Determinants and prediction of esterase substrate promiscuity patterns, ACS chemical biology 13 225–234.

[6] M. Yang, C. Fehl, K. V. Lees, E.-K. Lim, W. A. Offen, G. J. Davies, D. J. Bowles, M. G. Davidson, S. J. Roberts, B. G. Davis, (2018), Functional and informatics analysis enables glycosyltransferase activity prediction, Nature chemical biology 14 1109–1117.
